# Synthesis and Adsorption Property of SiO_2_@Co(OH)_2_ Core-Shell Nanoparticles

**DOI:** 10.3390/nano5020554

**Published:** 2015-04-03

**Authors:** Yongde Meng

**Affiliations:** Department of Chemistry, Hanshan Normal University, Chaozhou 521041, China; E-Mail: myd@hstc.edu.cn; Tel.: +86-768-2318680; Fax: +86-768-2318681

**Keywords:** core-shell, adsorption property

## Abstract

Silica nanoparticles were directly coated with cobalt hydroxide by homogeneous precipitation of slowly decomposing urea in cobalt nitrate solution. The cobalt hydroxide was amorphous, and its morphology was nanoflower-like. The BET (Brunauer-Emmett-Teller) surface area of the core-shell composite was 221 m^2^/g. Moreover, the possible formation procedure is proposed: the electropositive cobalt ions were first adsorbed on the electronegative silica nanoparticles surface, which hydrolyzed to form cobalt hydroxide nanoparticles. Then, the cobalt hydroxide nanoparticles were aggregated to form nanoflakes. Finally, the nanoflakes self-assembled, forming cobalt hydroxide nanoflowers. Adsorption measurement showed that the core-shell composite exhibited excellent adsorption capability of Rhodamine B (RB).

## 1. Introduction

In the past decade, research on core-shell nanoparticles has become an active field due to their chemical property, physical property, and potential applications in cell emerging [1,2], catalysis [3], energy storage [4], and drug delivery [5]. Owing to its uniform size and high stability, silica nanoparticle is often used as a template to create core-shell composite by coating with organic or inorganic material [6,7]. Among these, silica nanoparticles covered by a layer of cobalt-based nanostructures are of particular interest because these core-shell nanoparticles can bind the properties of a cobalt-based shell and the highly chemically stable core. Up to now, many core-shell composites with silica cores and cobalt-based shells have been synthesized, such as silica@cobalt [8], silica@cobalt/cobalt oxide [9], silica@cobalt ferrite [10] and silica@silica-cobalt oxide [11]. However, to the best of our knowledge, there is no report on the fabrication of core-shell nanoparticle with silica core and amorphous cobalt hydroxide nanoflowers shell.

Cobalt hydroxides are of interest due to their unique properties [12]. In particular, they have attracted intensive research efforts for electrochemical capacitors [13], electrodes for batteries [14], and electrocatalysts [15]. Cobalt hydroxide nanostructures with various morphologies, such as nanocones [16], nanoplates [17], nanodiscs [18], nanorods, nanosheets [19], nanoneedles [20], nanowires [21], nanoflake-like [22,23,24,25,26] and butterfly-like structures [27] have been fabricated by several methods.

Rhodamine B (RB) is one of the most important xanthenes dyes, which is used in a variety of applications such as paper and dye laser. It is also a common organic pollutant due to its toxicity. Hence, removal of RB is desired with regard to the purification of dye effluents.

In the present study, we have reported a synthesis of a core-shell composite with a silica core and an amorphous cobalt hydroxide nanoflower shell by homogeneous precipitation of slowly decomposing urea in cobalt nitrate solution. Moreover, its formation mechanism and adsorption performance as adsorbent for RB have also been investigated in this study.

## 2. Results and Discussion

The pH value in the solution has an effect on the hydrolysis and condensation of tetraethyl orthosilicate (TEOS). The formation of silica nanoparticles was carried out by the hydrolysis and condensation of TEOS in a mixture of ethanol-water-ammonia at a pH value of 9.4. The average size of as-prepared silica nanoparticles is *ca.* 200 nm. The silica nanoparticles were used as substrates, and the cobalt hydroxide was formed on the surface of silica nanoparticles by homogeneous precipitation of slowly decomposing urea in cobalt nitrate solution. Urea is a water-soluble compound. It decomposes to release ammonia and carbonate ions at 80 °C. Then, ammonia is hydrolyzed to produce the OH^−^ anions, which react with Co^2+^ to induce the formation of cobalt hydroxide.

**Figure 1:**
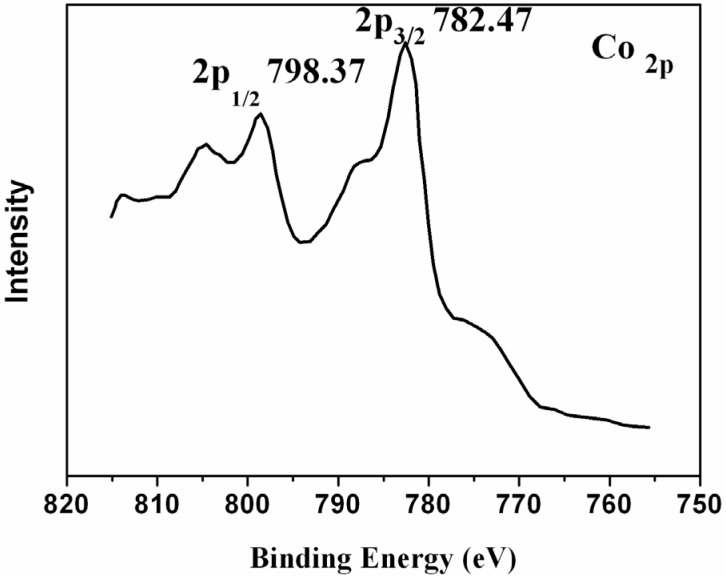
X-ray photoelectron spectra (XPS) pattern of silica/cobalt hydroxide nanocomposite.

As shown in Figure 1, the appearance of characteristic Co2p peaks at 798.37 eV and 782.47 eV and the separation of 15.9 eV between Co2p_1/2_ and Co2p_3/2_ confirm that the Co mainly exists as cobalt hydroxide [28]. Chemical synthesis of cobalt hydroxide in a simple precipitation reaction leads to auto-oxidation of Co^2+^ under high pH value [29]. In the present study, homogeneous precipitation by slowly decomposing urea in cobalt nitrate solution at low reaction temperature was chosen to avoid the high pH condition. The absence of trivalent cations (Co^3+^) on the silica surface was also checked by reacting with excess ferrosi sulfate and back titrating the excess ferrosi sulfate with standard potassium dichromate. Comparison with a blank titration confirms the absence of any trivalent ions (Co^3+^) on the silica surface. On the basis of above results, it is concluded that the shell in composite is mainly made up of cobalt hydroxide.

**Figure 2:**
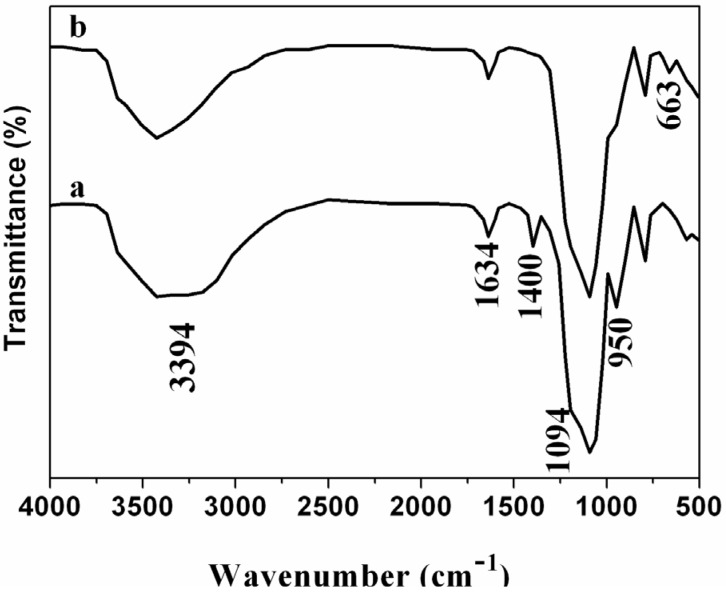
Fourier transform infrared (FT-IR) spectra of parent (**a**) Silica nanoparticles and (**b**) Silica/cobalt hydroxide nanocomposite.

Fourier transform infrared (FT-IR) spectrum of the obtained silica nanoparticles (Figure 2a) shows two strong bands at ~3393 cm^−1^ and ~1634 cm^−1^, which are due to the adsorbed water. The broad and intensive band of 3393 cm^−1^ is assigned to the O–H stretching vibration of water molecules, and the peak around 1634 cm^−1^ corresponds to the deformation vibration of O–H binds in water molecules. The absorptions at 1094 cm^−1^ and 950 cm^−1^ are assigned to the Si–O and Si–OH vibrations, respectively. The absorption at 1400 cm^−1^ is assigned to the residual NH_4_^+^ adsorbed on the silica surface [30]. No characteristic absorptions of NO_3_^−^ (1384 cm^−1^) and CO_3_^2−^ (1476 cm^−1^ and 836 cm^−1^) anions are observed in the FT-IR spectrum of the composite (Figure 2b), indicating cobalt hydroxide on the surface of silica nanoparticles instead of cobalt-hydroxide-salts. Compared to the FT-IR spectrum of silica nanoparticles, the absorption of NH_4_^+^ disappears in that of composite. This is due to NH_4_^+^ on the surface of silica exchanged by highly charged Co^2+^ in the coating process. According to previous reports on cobalt hydroxide, pure cobalt hydroxide is generally crystalline. However, the cobalt hydroxide on the surface of silica nanoparticles is amorphous from the corresponding X-ray diffraction (XRD) measurement (Figure S1), indicating that the cobalt hydroxide might contain some impurities [31]. In order to discern the impurities, the FT-IR analysis was employed to determine the impurities. In Figure 2b, a weak band shown at 663 cm^−1^ is attributed to the stretching vibration of Co(II)–O, which comes from cobalt oxides (CoO) [32]. The cobalt oxides were generated during the preparation of cobalt hydroxide [33]. Based on the FT-IR analysis and the XRD measurement, it is confirmed that the product contains some amorphous cobalt oxides, which lead to the formation of amorphous cobalt hydroxide.

**Figure 3:**
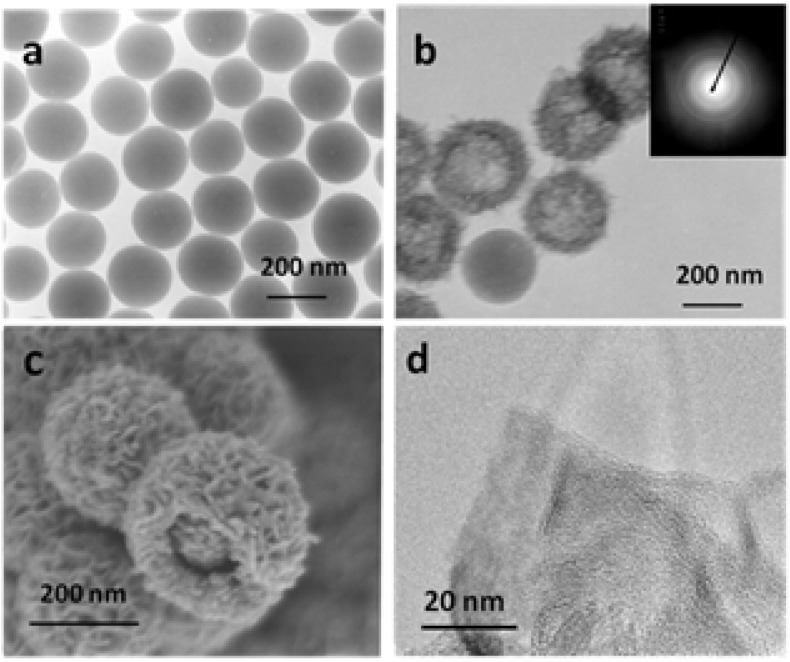
(**a**) Transmission electron microscope (TEM) image of silica nanoparticles; (**b**) TEM image of silica/cobalt hydroxide nanocomposite; (**c**) Field-emission scanning electron microscopy (FE-SEM) images of silica/cobalt hydroxide nanocomposite; and (**d**) High-resolution transmission electron microscope (HR-TEM) of cobalt hydroxide nanoflake. The insert in (**b**) corresponds to the selected area electron diffraction (SAED) pattern.

Figure 3a shows the transmission electron microscope (TEM) image of as-prepared silica nanoparticles. The silica nanoparticles are relatively uniform with smooth surfaces. The average size of silica nanoparticles is *ca.* 200 nm. After the coating process, it is clearly seen that the silica core is coated with uniform cobalt hydroxide shell due to one uncoated silica particle (Figure 3b). The thickness of cobalt hydroxide shell is *ca.* 60 nm. It is also confirmed to obtain the core-shell nanostructure from the field-emission scanning electron microscopy (FE-SEM) image (Figure 3c). High-resolution transmission electron microscope (HR-TEM) image in Figure 3d shows that the cobalt hydroxide is nanoflake-like in morphology, and amorphous in nature, which is consistent with the corresponding selected area electron diffraction (SAED) measurement (Figure 3b insert). The energy dispersive X-ray spectra (EDS) analysis shows the presence of Si, O and Co elements with a Co/Si atomic ratio of 1:4.4 in the silica/cobalt hydroxide composite, no N element is detected, indicating that the composite is free of NO_3_^−^ or NH_4_^+^ ions (Figure S2).

To reveal the formation mechanism of silica/cobalt hydroxide core-shell composite, the zeta potential was tracked during the depositing of the cobalt hydroxide shell. The zeta potential value of silica nanoparticles in the water is −24 mV, indicating negative charging of the silica nanoparticles in the system. After the addition of the cobalt nitrate, the zeta potential value of silica nanoparticles increased up to −10 mV, which resulted from the adsorption of the positively charged cobalt ions on the surface of silica nanoparticles via the electrostatic attraction. When the temperature was elevated to 80 °C, with the urea decomposing slowly, the pH value of the solution increased, and cobalt hydroxide would be firstly formed on the surface of silica nanoparticles by the hydrolysis of cobalt ions due to the high concentration. After the silica nanoparticles were covered with cobalt hydroxides, the zeta potential value of the composites increased up to −2.2 mV, demonstrating the formation of the core-shell nanostructure in that the silica/cobalt hydroxide nanoparticles behaved as one system rather than two separate species. The most important factor during the coating process is the stability of the initial seed suspension. The obtained silica nanoparticle is stable and negatively charged in water. The positively charged cobalt ion is adsorbed on the silica nanoparticle surface, and hydrolyzed to form cobalt hydroxide. This made it possible to form cobalt hydroxide shell directly without the help of stabilizer, surfactant, or coupling agent [34].

**Figure 4:**
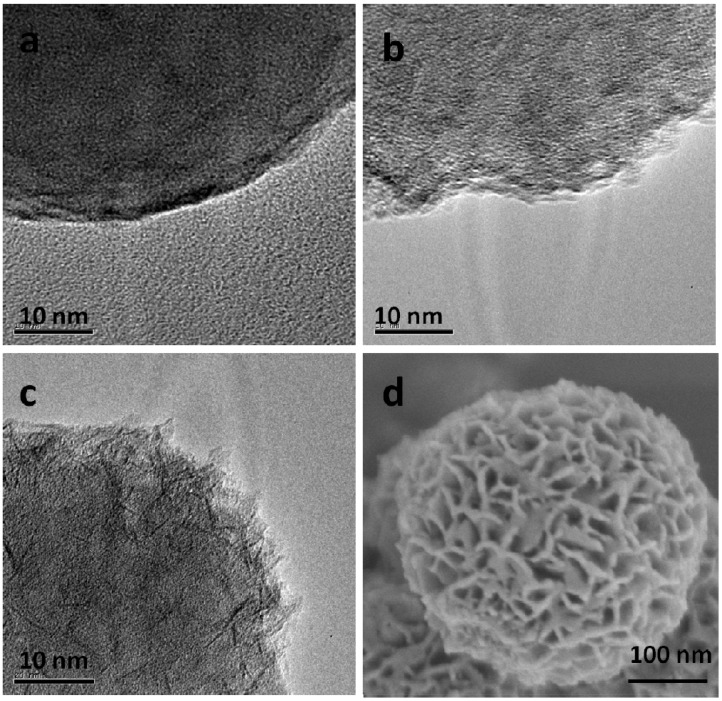
(**a**) HR-TEM image of sample at the beginning of reaction; (**b**) HR-TEM image of sample after 30 min of reaction; (**c**) HR-TEM image of sample after 90 min of reaction; (**d**) FE-SEM image of sample after 120 min of reaction.

**Figure 5:**
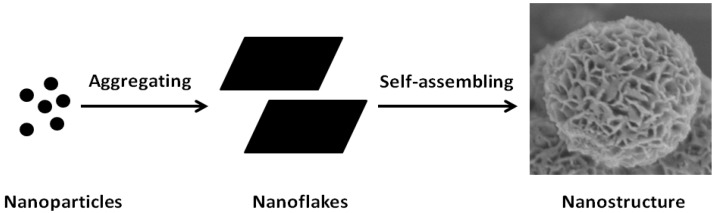
Proposed formation process of cobalt hydroxide nanoflakes.

To investigate the formation mechanism of the silica/cobalt hydroxide nanoparticles, time-dependent experiments were performed to gain insight into the formation process of cobalt hydroxide nanoflakes. Samples were collected at different stages from the reaction mixture once the temperature was elevated to 80 °C. Their morphology was subjected to both HR-TEM and FE-SEM investigations. Before coating process, the silica nanoparticles owned smooth surface (Figure 4a). After 30 min, the surface of the silica nanoparticle was observed to be coated with aggregates of nanoparticles with the size of 1 nm (Figure 4b). Nanoflakes were found to exist on the surface of silica nanoparticle after 90 min of reaction (Figure 4c). At 120 min, the silica nanoparticle was covered with the aggregates of nanoflakes (Figure 4d), which remained unchanged with the subsequent reaction. Based on the above observations, the formation process of the silica/cobalt hydroxide nanostructure was proposed as follows. First, the cobalt hydroxide nanoparticles were formed at the beginning stage. Then, the nanoparticles were aggregated to form the nanoflakes. Finally, the nanoflakes self-assembled, forming cobalt hydroxide nanostructures in order to minimize the surface energy of nanoflakes [35]. The formation mechanism is illustrated in Figure 5.

In previous reports, the homogeneous precipitation of cobalt salts with urea usually generated the cobalt—Hydroxide-salts in the shape of nanorods or needles. The anions were intercalated into the interlayer spacing to restore charge neutrality [36]. As an example, nanorods of cobalt-basic-carbonate were obtained by the homogeneous precipitation of cobalt nitrate with urea. However, in the presence of negatively charged core, the homogeneous precipitation of cobalt salt with urea generated amorphous cobalt hydroxide nanoflakes on the surface of silica nanoparticle. It is due to the fact that no CO_3_^2−^ and NO_3_^−^ ions can be adsorbed on the silica nanoparticle surface because of electrostatic repulsion between silica nanoparticle and anions. So it is reasonable that the anions (CO_3_^2−^ or NO_3_^−^) cannot be incorporated into the cobalt hydroxide to form cobalt-hydroxide-salts on the surface of silica nanoparticle. Therefore, this study provides a novel route to fabricating amorphous cobalt hydroxide nanoflakes.

**Figure 6:**
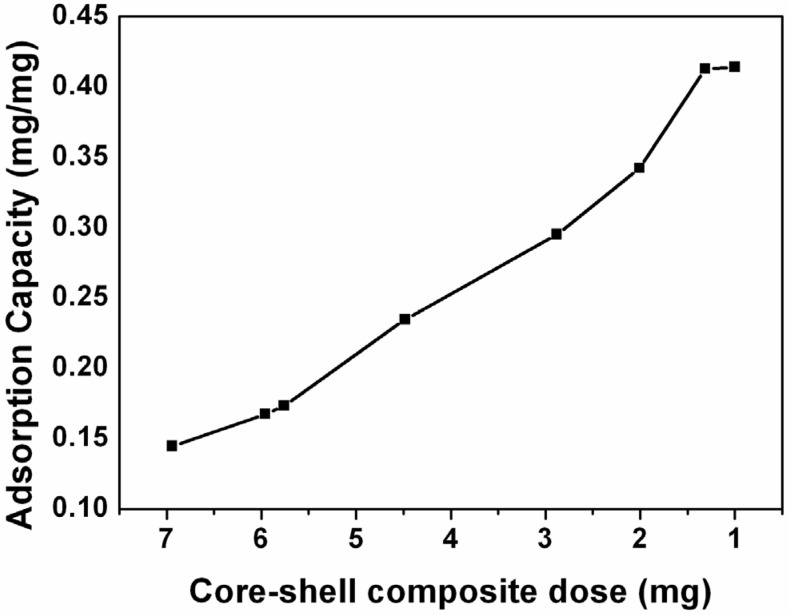
Adsorption capability of silica/cobalt hydroxide nanostructures.

It is believed that amorphous cobalt hydroxide nanoflakes have large surface area. The N_2_ adsorption-desorption isotherm of the core-shell composites was investigated. The results show that the BET surface area of the silica/cobalt hydroxide core-shell nanoparticle is 221 m^2^/g, and the most probable pore size is *ca.* 4.1 nm which originates from the self-assembly of the cobalt hydroxide nanoflakes (Figure S3). On the basis of its large surface area, it is predicted that negatively charged composite should have adsorption ability of cationic dyes. The RB was selected for adsorption test, as it is hazardous in wastewater. The effect of contact time for the adsorption of RB from the solution was studied. It is found that 97.4% of RB is adsorbed within 15 min, and 98% of RB is adsorbed after 30 min (Figure S4). The results show that RB uptake is rapid for the first 15 min, then proceeds at a slower rate, and finally attains equilibrium. The effect of core-shell composite dosage on the adsorption capacity was also investigated. It is found that the adsorption capacity increases from 0.14 mg/mg to 0.41 mg/mg while the core-shell composite dose decreases from 7 mg to 1 mg. The maximum adsorption capacity of composite for RB is 0.41 mg/mg (Figure 6). The results indicate that the core-shell composite has higher adsorption capability than those from previous papers on sorption of phosphate on cobalt hydroxide due to its high specific surface area [37].

## 3. Experimental Section

### 3.1. Synthesis

All reagents were of analytical grade and were used as received without further purification. The silica nanoparticles were synthesized by using the following procedure. In the mixture of 200 mL absolute ethanol, 18 mL deionized water and 6 mL ammonia, 1 mL tetraethylorthosilicate (TEOS) was added by drops under stirring. The molar ratio of CH_3_CH_2_OH**/**H_2_O**/**NH_3_/TEOS in the system was 3434**/**1000**/**77.6**/**2.1. The solution reacted for 2 h under stirring at the room temperature, and the precipitate was centrifugally separated from the suspension, and washed with absolute ethanol and water for three times. To fabricate the core/shell composite, 0.01 g of silica was ultrasonically dispersed in 100 mL deionized water to form a suspension, and then 0.3 g of urea (CO(NH_2_)_2_) and 0.15 g of cobalt nitrate (Co(NO_3_)_2_·6H_2_O) were added under stirring. The suspension was heated to 80 °C, and refluxed at that temperature for 2 h. Then, the sample was separated by centrifugation to obtain a pink precipitate. pH 6 was determined in the system at the end of the reaction.

### 3.2. Adsorption of RB

Solution of RB (50 mg/L) was prepared by dissolving the requisite amount of RB in distilled water and making up the desired volume. To study the effect of contact time for the adsorption of RB from the solution, 7 mg of core-shell composite was added into 20 mL RB solution (50 mg/L) at different time intervals (15–30 min). The sample was centrifuged to separate the solid particles, and the RB concentration in the supernatant liquid was determined using the Lambda-35 UV-VIS spectrometer at 552 nm. Percentage RB adsorption = (original amount of RB − Remaining amount of RB)/original amount of RB × 100%. The effect of core-shell composite dose on the adsorption capacity was studied by agitating 20 mL RB solution (50 mg/L) with a range of core-shell doses from 1 to 7 mg for 30 min. the sample was centrifuged to separate the solid particles, and the RB concentration in the supernatant liquid was determined using the Lambda-35 UV-VIS spectrometer at 552 nm. The adsorption capacity of composite for RB was calculated based on the following equation: The adsorption capacity (mg/mg) = (original amount of RB − Remained amount of RB)/amount of composite.

### 3.3. Characterization

XRD patterns were recorded using an X-ray diffractometer (Rigaku D/Max 2200PC, Japan Rigaku Corporation, Tokyo, Japan) with a graphite monochromator and CuKα radiation (λ = 0.15418 nm). A TEM (Model H-800, Hitachi, Tokyo, Japan) and a HR-TEM (JEOL-2010, JEOL Japan Electronics Co., Ltd, Tokyo, Japan) were applied to observe the morphology and microstructure of the samples. Surface image was characterized with FE-SEM (JEOL JSM-6700F, JEOL Japan Electronics Co., Ltd, Tokyo, Japan). The valence state of Co on silica surface was characterized by XPS (KRATOS, Kanagawa, Japan). The infrared (IR) spectra were measured on a FT-IR (Nicolet 5DX, Nicolet, Natus Neurology, Middleton, WI, USA) by using KBr pellet technique. The zeta potential was measured using a Zeta Meter (DXD-II, Jiangsu optical instrument corporation, Nanjing, China). N_2_ adsorption-desorption data were recorded on QUADRASORB SI (Quantachrome Instruments, Boynton Beach, FL USA) at liquid nitrogen temperature (*T* = −196 °C).

## 4. Conclusions

In summary, the amorphous cobalt hydroxide depositing on silica nanoparticle surface has been achieved by homogeneous precipitation method. The thickness of cobalt hydroxide shell is *ca.* 60 nm. The morphology of cobalt hydroxide is nanoflake-like. The cobalt hydroxide nanoflakes are formed by an aggregating mechanism. This synthetic strategy provides a novel route to amorphous cobalt hydroxide nanoflakes. The SiO_2_/Co(OH)_2_ composite exhibits excellent adsorption capability of RB due to its large specific surface area.

## Supplementary Materials

Supplementary materials can be accessed at: http://www.mdpi.com/2079-4991/5/2/554/s1.
