# Supplementary material for: Synthesis and Adsorption Property of SiO2@Co(OH)2 Core-Shell Nanoparticles
Source: Nanomaterials (Basel). 2015 Apr 3;5(2):554–64. doi: 10.3390/nano5020554 (PMC5312898; doi:10.3390/nano5020554)
Supplement: Supplementary file 1 [file nanomaterials-05-00554-s001.pdf]

Supplementary Materials

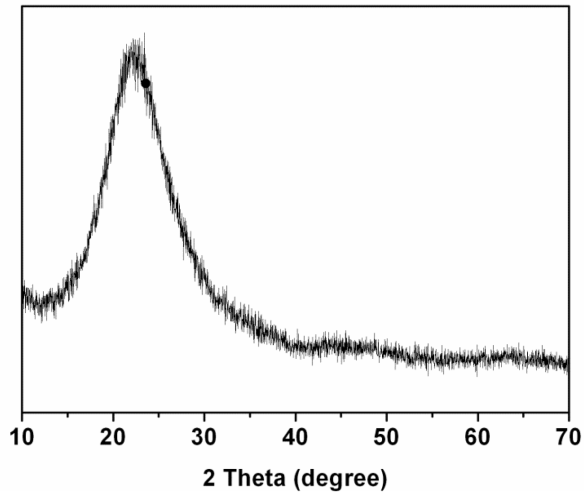

Figure S1. X-ray diffraction (XRD) pattern of core-shell composite.

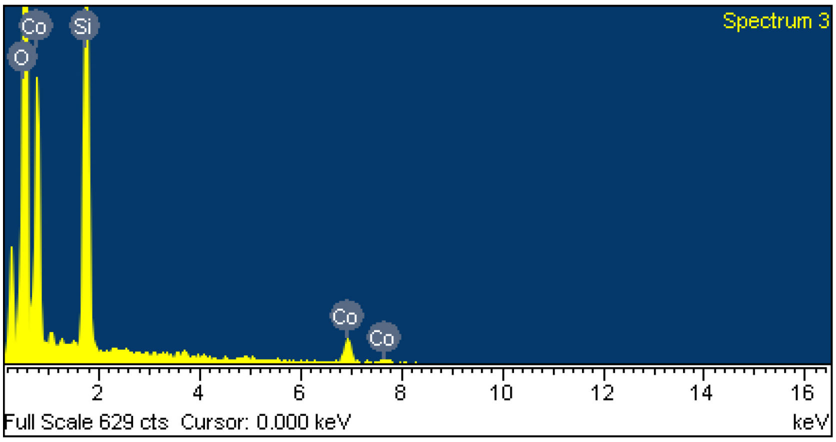

Figure S2. Energy dispersive X-ray spectra (EDS) pattern of core-shell composite.

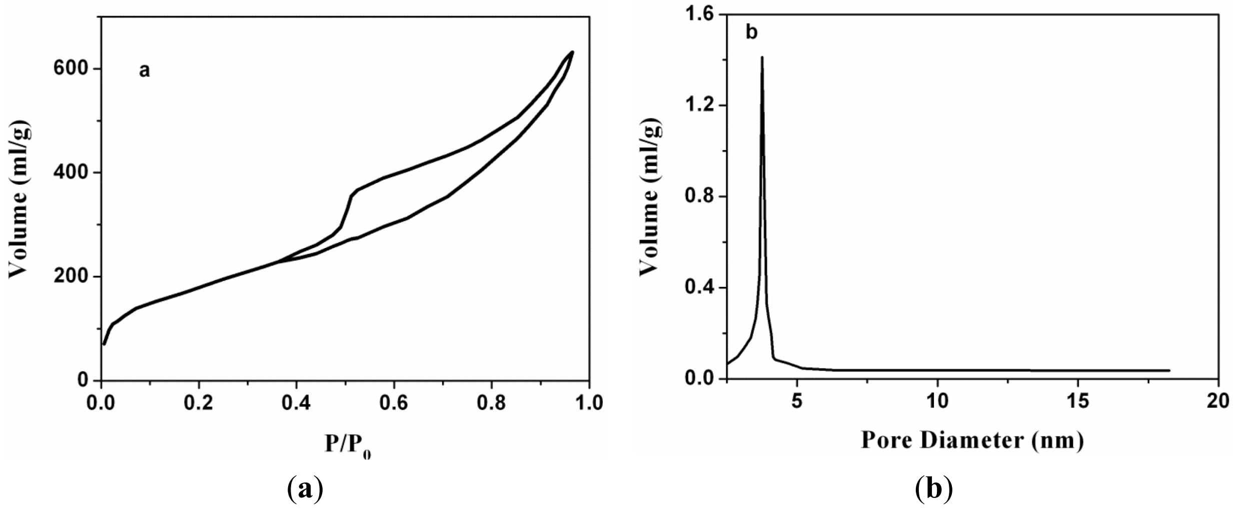

Figure S3. N_2 adsorption-desorption isotherm (**a**) and pore-size distribution (**b**) of core-shell composite.

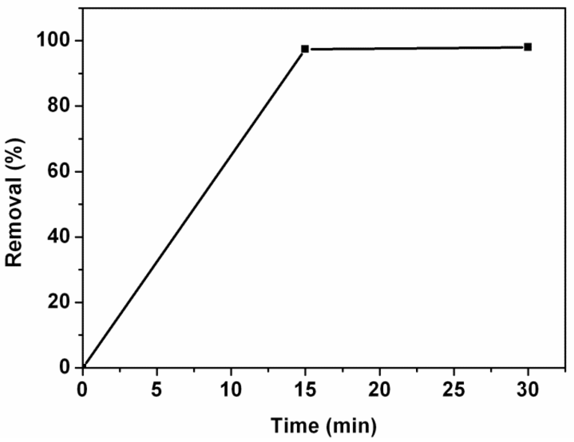

Figure S4. Effect of time on percent removal of Rhodamine B (RB).

© 2015 by the author; licensee MDPI, Basel, Switzerland. This article is an open access article distributed under the terms and conditions of the Creative Commons Attribution license (http://creativecommons.org/licenses/by/4.0/).
